# [Corrigendum] Suppression of SIPA‑1 expression may reduce bladder cancer invasion and metastasis via the downregulation of E‑cadherin and ZO‑1

**DOI:** 10.3892/etm.2022.11622

**Published:** 2022-09-23

**Authors:** Ping Zhang, Xinghuan Wang

Exp Ther Med 11:213–217, 2016; DOI: 10.3892/etm.2015.2891

Subsequently to the publication of the above article, an interested reader drew to the authors’ attention that Figs. 2 and 3 in their paper were published with errors that arose inadvertently during the assembly of these figures. In Fig. 2B, there was an apparent overlap in data comparing between the ‘BIU-87, 24 h’ and ‘Control BIU-87, 24 h’ data panels, whereas in Fig. 3A, there was an apparent overlapping section of data comparing between the ‘Invasion, T24’ and ‘Invasion, Control T24’ data panels. However, after having consulted their original data, the authors realized where the errors occurred in assembling these figures.

New versions of Figs. 2 and 3, showing replacement data for the experiments portrayed in the affected figure parts (Figs. 2B and 3A), are shown on the next page. Note that the revised data shown for these figures do not affect the overall conclusions reported in the paper. All the authors agree with the publication of this corrigendum; furthermore, they apologize to the Editor of *Experimental and Therapeutic Medicine* and to the readership for any inconvenience caused.

## Figures and Tables

**Figure 2:**
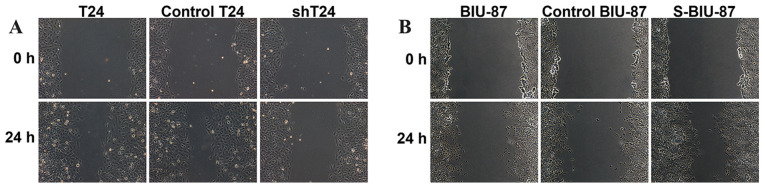
Wound and Transwell assays for T24 and BIU-87 cell migration and invasion. The metastasis of bladder cancer cells and transfected cells was measured using (A and B) wounding experiments. shT24, T24 cells transfected with short hairpin RNA targeting SIPA-1; S-BIU-87, BIU-87 cells transfected with the SIPA-1 gene. SIPA-1, signal-induced proliferation-associated protein 1.

**Figure 3:**
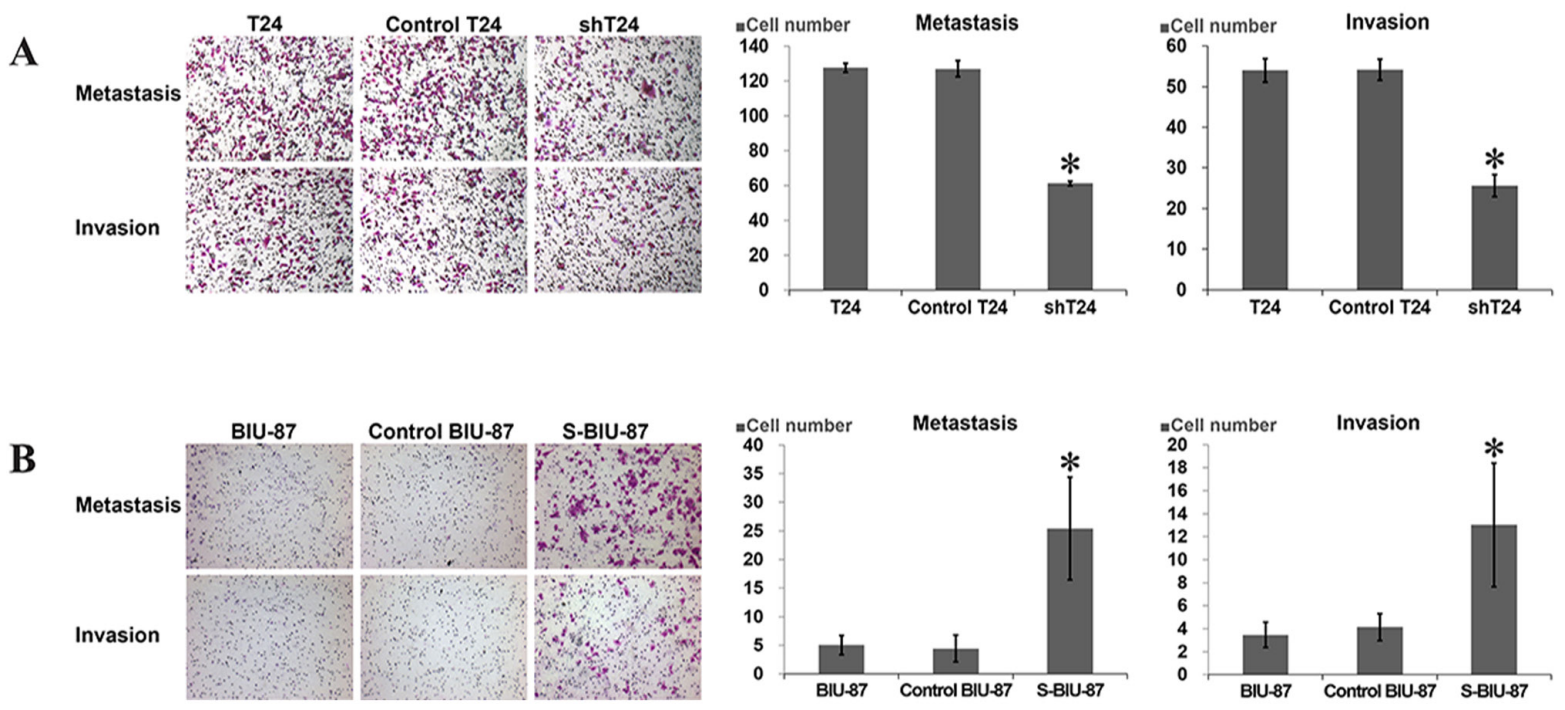
(A and B) The invasion of these cells was measured using Transwell assays with Matrigel. Results are the mean ± standard deviation for six independent experiments; ^*^P<0.05 vs. control and T24/BUI-87. shT24, T24 cells transfected with short hairpin RNA targeting SIPA-1; S-BIU-87, BIU-87 cells transfected with the SIPA-1 gene. SIPA-1, signal-induced proliferation-associated protein 1.

